# Facile synthesis and properties of a cation exchange membrane with bifunctional groups prepared by pre-irradiation graft copolymerization

**DOI:** 10.1039/c8ra03472a

**Published:** 2018-07-19

**Authors:** Jianhua Zu, Fangdong Tang, Linfeng He, Lingxiao Fu

**Affiliations:** School of Nuclear Science and Engineering, Shanghai Jiao Tong University Shanghai 200240 China zujianhua@sjtu.edu.cn; Shanghai Institute of Measurement and Testing Technology Shanghai 201203 China

## Abstract

A new type of a cation exchange membrane named ETFE-*g*-poly(AA-*co*-SSS) with bifunctional groups was synthesized by a one-step method. Its preparation by an electron beam-induced pre-irradiation grafting method and the effects of reaction temperature, monomer concentration, pH value of the grafting solution, storage time and temperature of the irradiated poly(ethylene-*alt*-tetrafluoroethylene) (ETFE) films on the grafting yield were studied. A total concentration of 2 mol L^−1^ of monomers was found to be beneficial for acrylic acid (AA) and sodium styrene sulfonate (SSS) co-grafting onto the ETFE films. Infrared spectroscopic analysis of the grafted membrane confirmed the existence of sulfonate and carboxylic acid groups. The contact angle of the grafted membrane decreased from 94.3 to 46.7° with the increase in grafting yield. The higher the grafting yield, the faster the response and recovery rate with respect to humidity. AFM images showed that the diameter of the grafted chains on the surface of ETFE membranes was about 30 nm. The voltage of the grafted membrane was stable after 100 cycles of charge–discharge; thus, the prepared membranes have great potentials to be used as separators in secondary batteries.

## Abstract 2

Low-cost and anti-degradation ion-exchange membrane named ETFE-*g*-poly(AA-*co*-SSS) with bifunctional groups prepared by the pre-irradiation grafting method.

## Graphical abstract

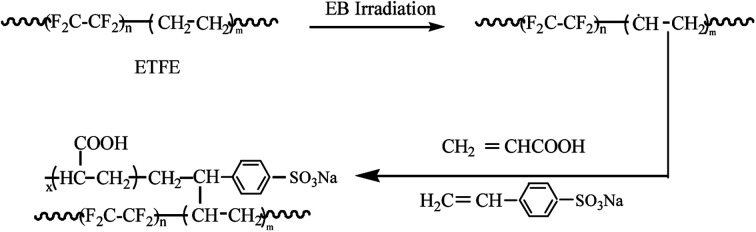

## 1. Introduction

Traditional cation exchange membranes are mostly prepared by physical blending or chemical copolymerization. Since 1971, the DuPont company has produced commercial ion exchange membranes named Nafion membranes with sulfonate groups. The membranes have low resistance and high stability, but their degradation rate is accelerated when the cathodic potential is relatively high.^1^ In addition, Nafion membranes also have the defects of high cost, low conductivity and poor resistance to methanol at high temperature.^2^ Radiation-induced graft polymerization is a promising method to introduce desirable properties onto polymers because of its simplicity in controlling the grafting parameters. Monomer or monomer mixtures with functional groups are grafted onto ready-made films so that the film shaping process can be excluded as the preparation begins with a polymer already in the film form.^3^ All membranes synthesized by the radiation-induced grafting technique have special advantages over other membranes prepared by blending or polymerization modification. Membranes prepared by this technique have wide applications in proton exchange membrane fuel cells,^4^ secondary batteries,^5,6^ and treatment of wastewater.^7^

Although various efforts have been made to prepare high-performance membranes for ion exchange,^8,9^ so far, only few radiation-grafted ion exchange membranes have been successfully applied in batteries or fuel cells for large-scale commercialization because of a few key challenges; these include (1) radiation degradation of polyolefin and monomer polymerization initiated by radicals produced by radiation, and (2) the high cost of certain starting materials and irradiation facility. In China, for commercial development of ion exchange membranes, the membranes are mainly prepared by copolymerization induced by a chemical initiator. Researchers engaged in the membrane manufacturing technology have a background in polymer chemistry. In the next 5 to 10 years, we strongly believe that with continuous research in this field, great progress will be made towards the manufacture of radiation-grafted cation membranes and their applications in secondary batteries or fuel cells.

We have reported that the addition of acid plays a significant role in radiation-induced grafting of vinyl monomers such as sodium styrene sulfonate (SSS) and acrylic acid (AA) onto polyethylene.^10^ Based on our research, Nasef *et al.* further prepared proton exchange membranes containing only sulfonic acid groups by adding an aqueous acid solution to the grafting mixture.^11^ However, membranes with –SO_3_H groups can swell in water to a great extent due to their strong hydrophilicity, whereas membranes containing –COOH groups, which are weakly acidic and less hydrophilic, swell to a lesser extent in water. Patent applications and research done by Henkensmeier *et al.* have demonstrated that membranes having two different ion-exchange groups perform better than membranes having one type of an ion-exchange group.^12,13^ Our attempt aims at synthesizing membranes having more than one type of ion-exchange group so as to achieve better performance in secondary batteries or humidity sensors. In our previous study, we carried out grafting of AA and SSS comonomers onto high density polyethylene (HDPE) by the pre-irradiation technique, and we studied the effects of various reaction parameters on the degree of grafting.^14,15^ We used electron spin-resonance spectroscopy to study the stability of radicals formed on HDPE and poly(ethylene-*alt*-tetrafluoroethylene) (ETFE) films by electron beam irradiation. The results indicated that the trapped radicals at the crystalline region of ETFE were more stable than the radicals of HDPE under the same absorption doses and storage conditions. A further improvement is expected by changing the matrix from HDPE to partially fluorinated ETFE, which is commercially available for approximately 3€ per m^2^. In addition, our research team has been devoted to the development of low-cost and anti-degradation ion-exchange membranes by radiation-induced grafting methods. To improve the stability of the modified polymer, a partially fluorinated film has been chosen as the polymer matrix.^16^ Several advantages over perfluorinated films are identified such as superior mechanical properties,^17^ faster grafting kinetics due to higher monomer compatibility and less radiation-induced damage.^18,19^

Shkolnik *et al.* reported the difficulties in direct grafting of sodium styrene sulfonate (SSS) onto fluoropolymers by radiation-induced grafting because of the incompatibility between the highly ionized sulfonic acid groups and its hydration sphere and the hydrophobic polymer matrix.^20^ Hence, a two-step grafting method was reported for the preparation of ion-exchange membranes bearing sulfonic acid groups.^21–24^ First, styrene or substituted styrene monomers was grafted onto the polymer films and then, the grafted membrane was immersed into chlorosulfonic acid or sulfuric acid at a higher temperature to introduce sulfonic groups into the benzene ring.^25–27^ The physical strength of the membranes deteriorated because of strongly acidic reaction conditions. Therefore, to simplify the preparation process and to improve the mechanical properties of the obtained membranes, we designed a co-monomer graft system to directly introduce –SO_3_H and –COOH groups onto fluorinated films by a one-step grafting method. Hence, a detailed study on the influence of grafting parameters was performed for the grafting of acrylic acid (AA) and SSS onto ETFE films. The introduction of both –COOH and –SO_3_H groups onto ETFE films further improved the swelling property and sensitivity to humidity of the grafted membranes.

## 2. Experimental

### 2.1 Materials

ETFE films of 50 μm were purchased from Nowofol GmbH (Siegsdorf, Germany). Acrylic acid (AA) was obtained from Sinopharm Chemical Reagent Co. Ltd. AA was purified by reduced pressure distillation to remove any inhibitors. Sodium styrene sulfonate (SSS) was bought from Zibo Jinyuelong Chemical Ltd and used without purification. Commercial battery separator was bought from Shanghai Shilong Hi-Tech. Ltd.

### 2.2 Equipment

A 2 MeV accelerator (GJ-2, Shanghai Xianfeng Electric Machinery Plant) was used to irradiate ETFE films. The structure and morphology of ungrafted and grafted membranes were characterized by FT-IR spectroscopy (IR200, Nicolit) and AFM (Multimode NanoscopeIIIa, Bruker), respectively. The pH value of the solution was determined with a pH meter (Seven compact™, Mettler Toledo Co., Ltd.).

### 2.3 Experimental method

#### 2.3.1 Grafting procedure

**Scheme 1:**
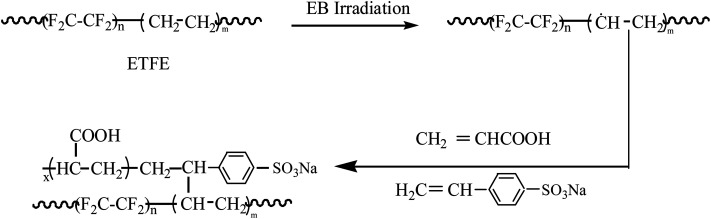
Illustration for preparation of a bifunctional cation exchange membrane.

ETFE films were cut into rectangles (length: 6 cm; width: 4 cm). All films were washed with acetone and then dried in a vacuum oven until a constant weight was obtained. Then, the ETFE films were packaged into self-sealing plastic bags, which were filled with high purity nitrogen. Irradiation of ETFE films was carried out on driving devices of an electron accelerator with an electron beam intensity of 1 mA. The irradiated films together with polypropylene non-woven fabrics were rolled into cylindrical shapes; then, the films were immersed into the grafting solution that was prepared at the given concentrations and deaerated by purging high purity nitrogen. The grafting reaction was carried out in a constant-temperature water bath for a fixed time. The preparation scheme is shown in Scheme 1.

#### 2.3.2 Measurement of total grafting yield (*G*_t_)

After the reaction was completed, the grafted membranes were removed from the grafting solution and washed with distilled water to remove homopolymers and copolymers attached to the membranes. After being dried in a vacuum oven at 40 °C until a constant weight, *G*_t_ was calculated as follows:
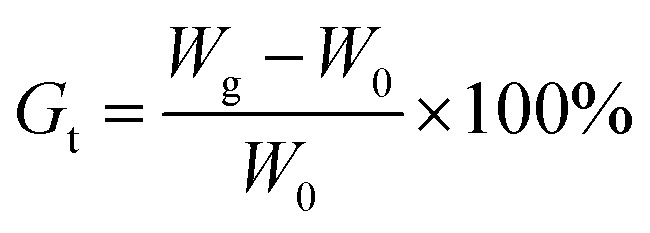
here, *W*_0_ and *W*_g_ are the weights of the ungrafted and grafted membranes, respectively. Before grafting, the thickness of the ETFE film was 50 μm. The thickness of the grafted membranes varied with *G*_t_. For example, the thickness was 128 μm for *G*_t_ = 98.3%.

#### 2.3.3 Measurement of grafting yield of SSS onto ETFE (*G*_s_)

The grafted membranes were immersed into 1 mol L^−1^ HCl solution, which was stirred with a magnetic stirrer until –SO_3_Na groups on the membranes transformed to –SO_3_H. After being removed from the HCl solution, the membranes were washed with distilled water until the pH of the distilled water was 7. The membranes were then placed in 5% NaCl solution for 24 hours while being stirred. H^+^ on the membranes exchanged with Na^+^ from the solution. –COOH on the grafted membranes ionized and generated H^+^, but this can be ignored because the other functional –SO_3_H groups on the grafted membranes were strong acids, and H^+^ obtained from the ionization of –SO_3_H could inhibit the ionization of –COOH. The replaced H^+^ was titrated with an NaOH standard solution. *G*_s_ can be calculated according to the following formula:
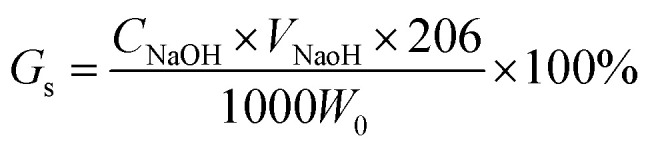
here, *C*_NaOH_ is the concentration of NaOH (mol L^−1^), and *V*_NaOH_ is the volume of NaOH (mL).

## 3. Results and discussion

### 3.1 The influence of reaction time on *G*_t_ and *G*_s_

**Fig. 1:**
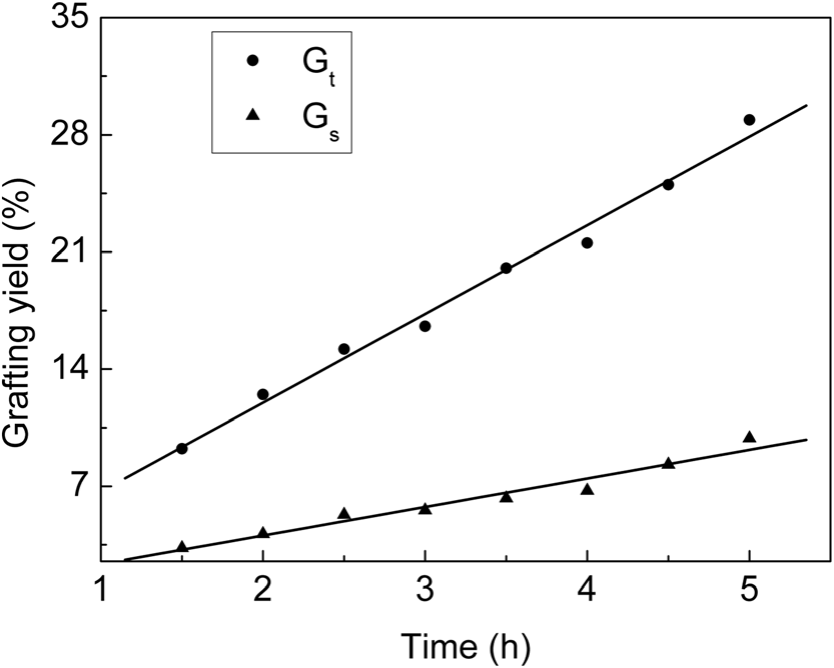
Variation of *G*_t_ and *G*_s_ with reaction time. Total monomer concentration: 2 mol L^−1^; molar ratio of AA to SSS: 1 : 1; reaction temperature: 60 °C; absorbed dose: 150 kGy.

From Fig. 1, we can see that *G*_t_ and *G*_s_ increase with the increasing grafting time at fixed monomer concentration and molar ratio. During the grafting time from 1.5 to 4.5 h, *G*_t_ increases linearly with the reaction time. At the beginning, the grafting reaction occurs on the surface of the ETFE membranes and is controlled by the monomeric diffusion rate. AA is grafted onto ETFE and then, the hydrophilicity of ETFE membranes improves; therefore, both AA and SSS react with the radicals formed on the graft chains. Due to the increase in membrane swelling and monomer diffusion rate, different thicknesses of the ETFE films can be finally introduced for the AA and SSS monomers.^28,29^

### 3.2 The influence of monomer concentration on *G*_t_

**Fig. 2:**
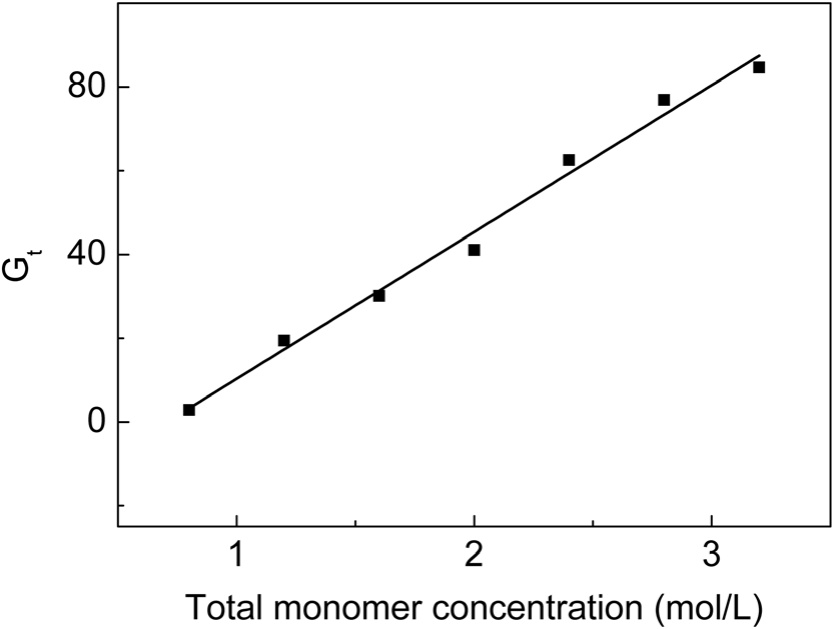
Variation of *G*_t_ with total monomer concentration. Absorbed dose: 150 kGy; molar ratio of AA to SSS: 1 : 1; reaction temperature: 60 °C; reaction time: 5 h.

From Fig. 2, we can see that *G*_t_ increases from 2.9 to 84.6% when the monomer concentration varies from 0.8 to 3.2 mol L^−1^ at a fixed molar ratio. High monomer concentrations accelerate the diffusion rate of the monomers into the ETFE matrix so that more monomers can react with the radicals formed on ETFE by irradiation. However, it was reported that the grafting yield increased with the increasing monomer concentration to a certain value after which the grafting yield dropped with the increasing monomer concentration.^30^ In our grafting system, on one hand, SSS has limited solubility in the grafting solution; on the other hand, the homopolymerization or the copolymerization rate of monomers is slower than the grafting rate when monomer concentrations are lower than 3.2 mol L^−1^.

### 3.3 The influence of reaction temperatures at different grafting times on *G*_t_

**Fig. 3:**
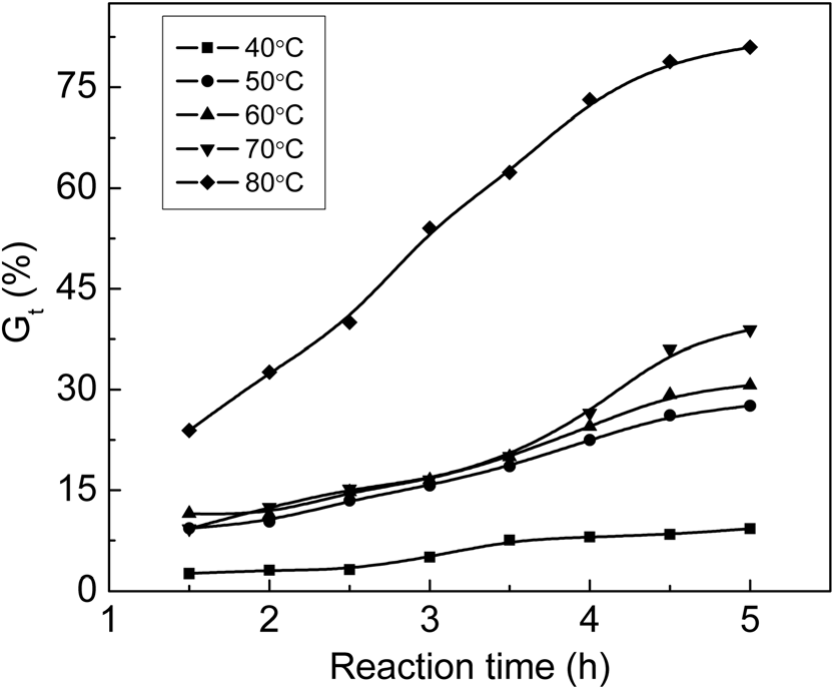
Variation of *G*_t_ with time at various temperatures. Absorbed dose: 150 kGy; molar ratio of AA to SSS: 1 : 1; total monomer concentration: 2 mol L^−1^.

Fig. 3 shows that *G*_t_ increases with an increase in temperature. *G*_t_ is less than 10% at 40 °C although the grafting reaction continues for 5 hours, which indicates that the diffusion rate of monomers to the ETFE matrix is very slow at 40 °C; therefore, the grafting reaction has long induction period under low temperature. When the grafting temperature is higher than 50 °C, *G*_t_ clearly increases with the increasing temperature due to greater membrane swelling and faster monomeric diffusion rate. After the grafting reaction is continued for 4.5 h, *G*_t_ almost levels off for all temperatures. After the introduction of hydrophilic –COOH and –SO_3_Na functional groups, the grafted membrane gradually swells in the grafting solution, which makes the graft chains highly movable within ETFE. In this case, the termination rate of the growing chains by mutual combination is accelerated.^31^ A similar tendency has also been reported for the grafting of styrene onto ETFE-based films.^32^

### 3.4 The influence of pH values of the grafting solution on *G*_t_

**Fig. 4:**
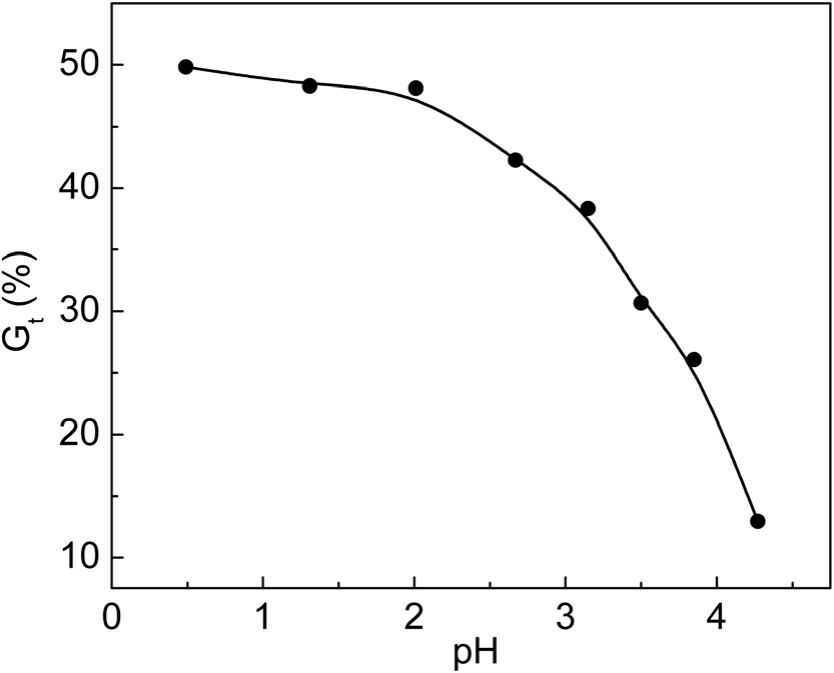
Variation of *G*_t_ with pH value. Total monomer concentration: 2 mol L^−1^; molar ratio of AA to SSS: 1 : 1; reaction temperature: 60 °C; absorbed dose: 150 kGy; reaction time: 3 h.

Additives such as acids and metal salts were used in the radiation grafting system. Acidic additives seemed to have a very important effect on the grafting yield. Different acids were added to the grafting solution to improve the grafting yield and lower the cost of the prepared functional materials. To enhance the grafting yield, the addition of inorganic acids is applicable to many grafting systems.^33,34^ The relationship between *G*_t_ and pH is shown in Fig. 4. The original pH value of the solution without adding HCl was 2.67. We obtained a pH value of 4.27 by dripping 0.1 mol L^−1^ NaOH solution into the grafting system. It was found that *G*_t_ was 49.8% at pH of 0.49, and it dropped to 12.9% when pH was 4.27. We found that the viscosity of the solution at pH = 0.49 was higher than that of the solution at pH = 4.27 when the grafted membranes were removed from the solution. The movement of the grafted chain with high molecular weight was restricted due to high viscosity of the grafting solution. Meanwhile, the monomers and their homopolymers with low molecular weights could freely diffuse and react with the growing grafted chains. The propagation rate of the grafted chains was faster than their termination rate; thus, *G*_t_ increased rapidly at pH = 0.49. The increase in grafting yield in simultaneous irradiation grafting systems by adding an inorganic acid was concluded based on the assumption that the addition of acid accelerated hydrogen abstraction by the monomers from nearby polymer molecules.^35^ For the pre-irradiation grafting system, research work done by Garnett indicated that the acid enhancement in the grafting yield was due to a partitioning effect.^36^

### 3.5 The influence of storage time of irradiated membranes on *G*_t_

**Fig. 5:**
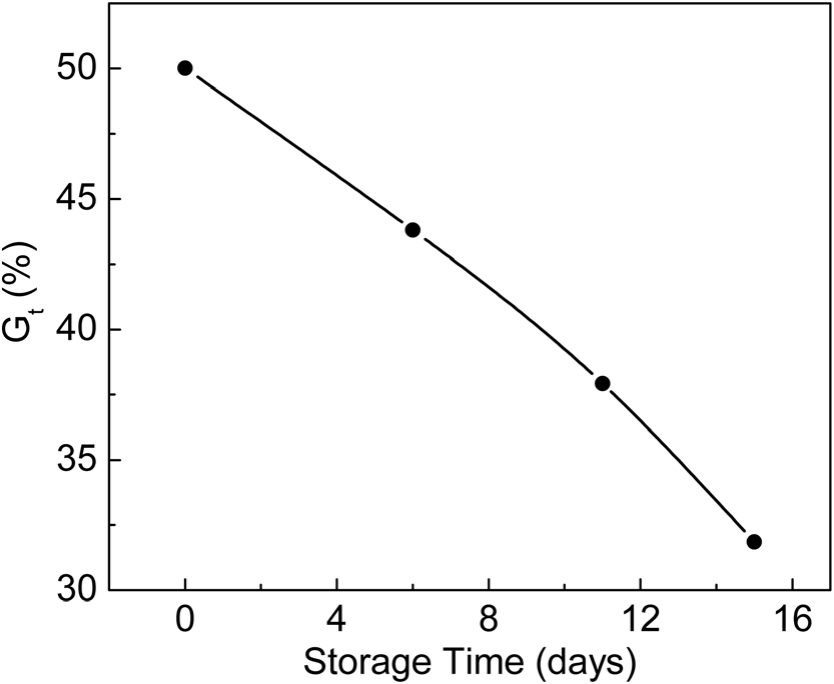
Variation of *G*_t_ with storage time. Absorbed dose: 150 kGy; molar ratio of AA to SSS: 2 : 1; total monomer concentration: 2 mol L^−1^; reaction time: 2 h.

ETFE films irradiated with an electron beam in a nitrogen atmosphere were stored at −19 °C for different storage times and reacted with monomers at 80 °C. The relationship between *G*_t_ and storage time is shown in Fig. 5. *G*_t_ was 50.0% when the grafting reaction was carried out immediately after irradiation. Under the same reaction conditions, the *G*_t_ values dropped to 43.8% and 31.9% during 6 and 15 days of storage times, respectively. The trapped radicals formed in the ETFE film decayed with longer storage times. The lower the storage temperature, the longer is the lifetime of the radicals. Gupta *et al.* found that the grafting yield of an FEP film remained almost unchanged after 118 days of storage time at a temperature of −60 °C.^37^

## 4. Characterization of grafted membranes

### 4.1 FTIR test of grafted membranes

**Fig. 6:**
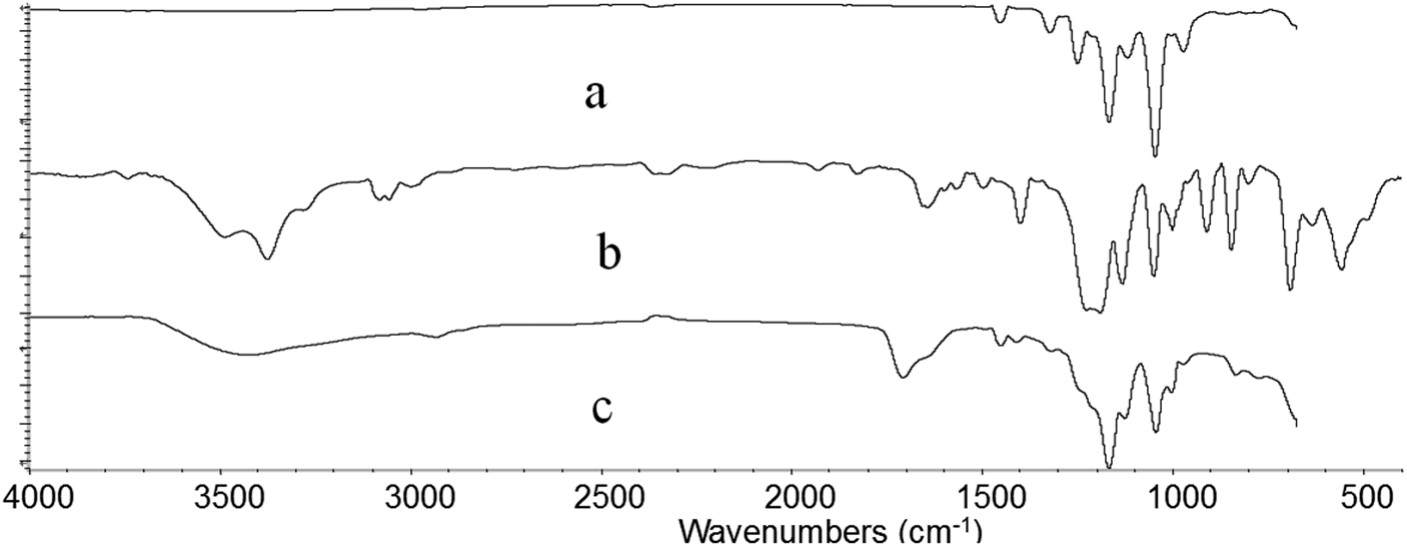
FTIR spectrum of irradiated ETFE film (a), ETFE-*g*-AA (b) and ETFE-*g*-AA-*co*-SSS (c).

Fig. 6 shows the FTIR spectra of irradiated ETFE film (a), ETFE film grafted with AA having 98.3% grafting yield (b) and ETFE film grafted with AA and SSS with 141% grafting yield (c). Comparing the spectrum (a) with (b), a broad absorption peak between 3200 and 3650 cm^−1^ is seen, which is a characteristic peak for –OH stretching vibrations of AA. The absorption peak at 1732 cm^−1^ corresponds to the C=O groups of AA. For the spectrum of ETFE-*g*-poly(AA-*co*-SSS) membrane (c) compared with spectrum (a), the absorption peak at 842 cm^−1^ is the characteristic peak of a substituent attached to the *para*-position of benzene ring. The peak at 1044 cm^−1^ is due to a symmetrical stretching vibration of S=O.^38^ All these new adsorption peaks indicate that AA and SSS have been successfully grafted onto the ETFE film.

### 4.2 AFM test of grafted membranes

**Fig. 7:**
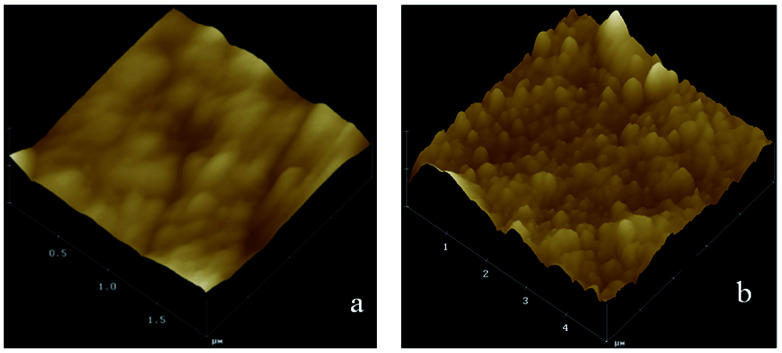
AFM images of irradiated ETFE (a) and grafted ETFE (b).

The surface roughness and topography of original ETFE and grafted membranes were tested by an Atomic Force Microscope (AFM) equipped with the nanoscope analysis software (Multimode NanoscopeIIIa) at room temperature. The AFM test was performed in a tapping mode, and a measurement of roughness of the 3-D surface was estimated. Fig. 7 shows AFM images for the surface morphology of the irradiated ETFE film (a) and the ETFE-*g*-poly(AA-*co*-SSS) membrane with *G*_t_ = 39.6% (b). Comparing the image (a) with (b), we inferred that the surface of the membrane became thicker and rougher because of the formation of grafted chains on the ETFE surface. The diameter of the grafted chains measured from the 3-D images was about 30 nm.

### 4.3 The influence of grafting yield on contact angles

**Fig. 8:**
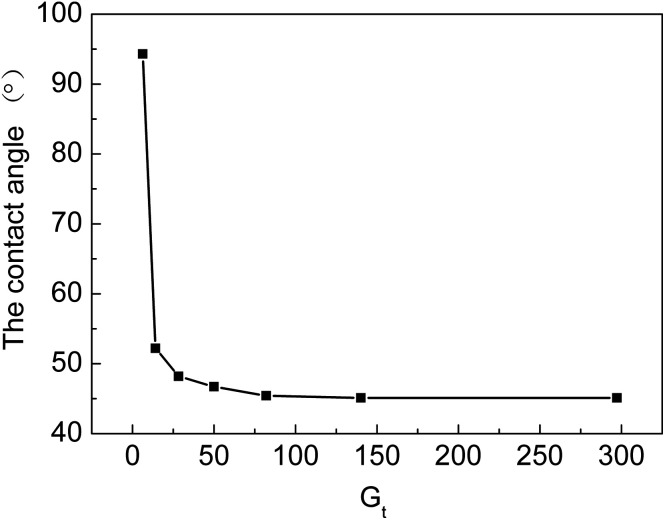
Relationship between *G*_t_ and water contact angle.

From Fig. 8, we can see that water contact angles first decrease with an increase in *G*_t_ and then, they almost level off with a further increase in *G*_t_. The contact angle decreases from 94.3 to 46.7° when *G*_t_ rises from 6.3% to 50%. At the beginning of the grafting reaction, the hydrophilic grafted chains, which contain –COOH and –SO_3_H groups, only cover part of the surface of the membranes. When *G*_t_ is higher than 50%, the grafting chains are uniformly distributed on the surface of the grafted ETFE, due to which the contact angle remains almost unchanged. When *G*_t_ is higher than 50%, the graft reaction mainly occurs at a different depth of ETFE films. The hydrophilicity of the cation exchange membranes has a significant effect on their ionic conductivity. If the grafting yield is low or only parts of the ETFE film are grafted, ion-exchange groups are distributed inhomogeneously on the membrane. Furthermore, the hydrophilicity of the membrane also influences other performance characteristics, for example, compatibility, dimensional stability and resistance to methanol at high temperature when the membrane is used in a proton exchange fuel cell. To prepare cation exchange membranes with long-term stability, hydrophilicity of the membrane should be optimized by changing the grafting conditions.

### 4.4 Electrochemistry performance of grafted membrane

**Fig. 9:**
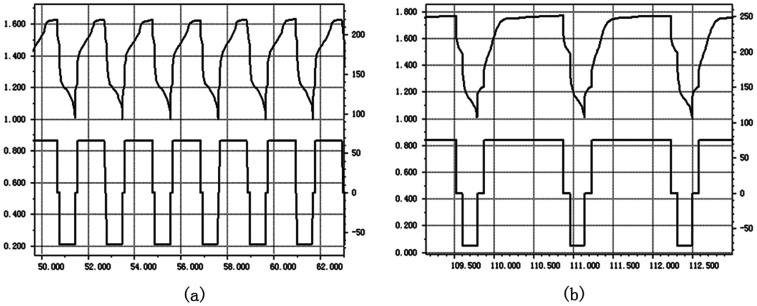
Electrochemistry spectra of grafted ETFE membrane (a) and commercial battery separator (b).

The grafted ETFE membrane with *G*_t_ = 135% and a commercial battery separator were separately assembled within batteries, and their electrochemistry performances were tested by charging and discharging cycles. The battery electrode was wrapped by a grafted membrane or a commercial battery separator and then soaked into 6 mol L^−1^ KOH solution. After activating for 8 h, the electrochemistry performance of the assembled batteries was tested. Fig. 9(a) and (b) show parts of the charge and discharge spectra of the grafted membrane and the commercial separator, respectively. The whole test was performed at an electric current of 66 mA for 200 h. For the grafted membrane, the charging voltage dropped from 1.66 V to 1.60 V. For the commercial separator, the charging voltage reduced from 1.83 V to 1.77 V. A lower charging voltage indicates that the resistance of the grafted membrane is lower than that of the commercial separator under the same test conditions. Low resistance is very important for grafted membranes used as battery separators, because the lower the film resistance, the greater the current when the battery is discharging. Furthermore, after 100 cycles of charging and discharging, the charging voltage was still relatively stable. Thus, the prepared ETFE-*g*-poly(AA-*co*-SSS) membranes have great potentials to be used as separators in secondary batteries.

### 4.5 The relationship between *G*_t_ and alkali absorption property

**Fig. 10:**
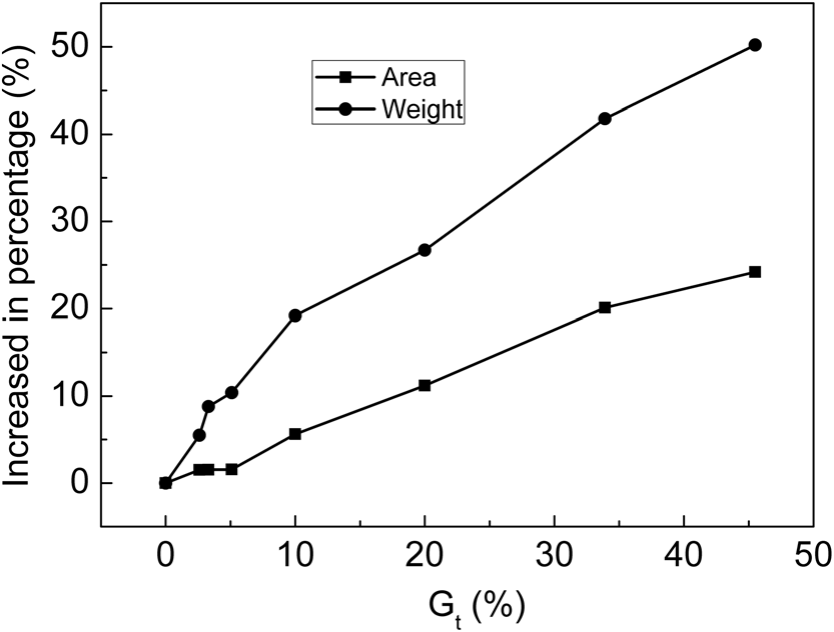
Variation of percentage increase in area and weight with *G*_t_.

The membranes with *G*_t_ = 0, 2.6, 3.3, 5.1, 10.0, 20.0, 33.9 and 45.5% were soaked in 30% NaOH solution for 24 h at 25 °C and then taken out. After water on the surface of membranes was wiped off, the area and weight of the membrane were measured. The relationship between the increase in the percentage of weight or area is shown in Fig. 10. After the membranes were swollen in 30% NaOH solution, the area and weight of the membranes clearly increased with increasing *G*_t_. Alkali absorption property is another key factor for evaluating the performance of ion-exchange membranes as separators of alkaline batteries. Because the membranes synthesized in this research contained both strong and weak acid groups when compared with membranes with only carboxylic acid groups, the alkali absorption rate was significantly improved.

### 4.6 Influence of environmental humidity on the resistance of grafted membranes

**Fig. 11:**
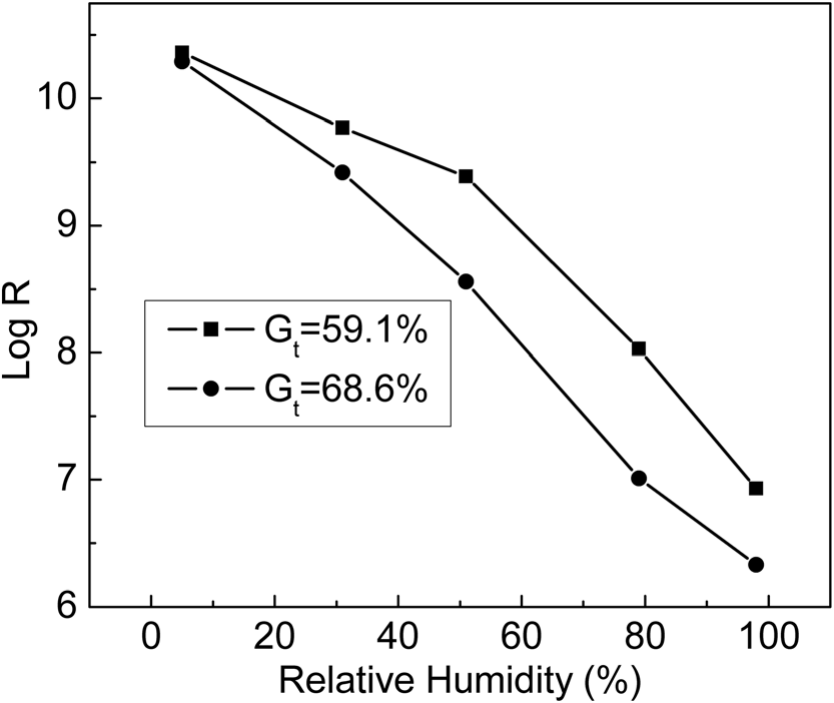
Relationship between log *R* and relative humidity at different *G*_t_.

The humidity sensitivity curves of ETFE-*g*-poly(AA-*co*-SSS) membranes with different *G*_t_ values were measured at 25 °C at the following humidity values: 5%, 31%, 51%,79%, and 98%. From Fig. 11, we can see that the resistance of the grafting membranes (*R*) decreased with the increase in environmental humidity. At the same humidity, the resistance of the membrane with *G*_t_ = 68.6% was lower than that of the membrane with *G*_t_ = 59.1%. When *G*_t_ increased, more –COOH and –SO_3_Na groups grafted onto ETFE membranes were ionized at high humidity. When the humidity was 98%, the membrane resistance dropped by 4 orders of magnitude compared with that at humidity of 5%. All the results indicated that the grafting membranes with hydrophilic –COOH and –SO_3_Na groups responded quickly to the change in humidity. Therefore, the prepared membrane with bifunctional groups can be used as a humidity sensitive material of a humidity sensor.

### 4.7 Response and recovery curves of grafted membranes

**Fig. 12:**
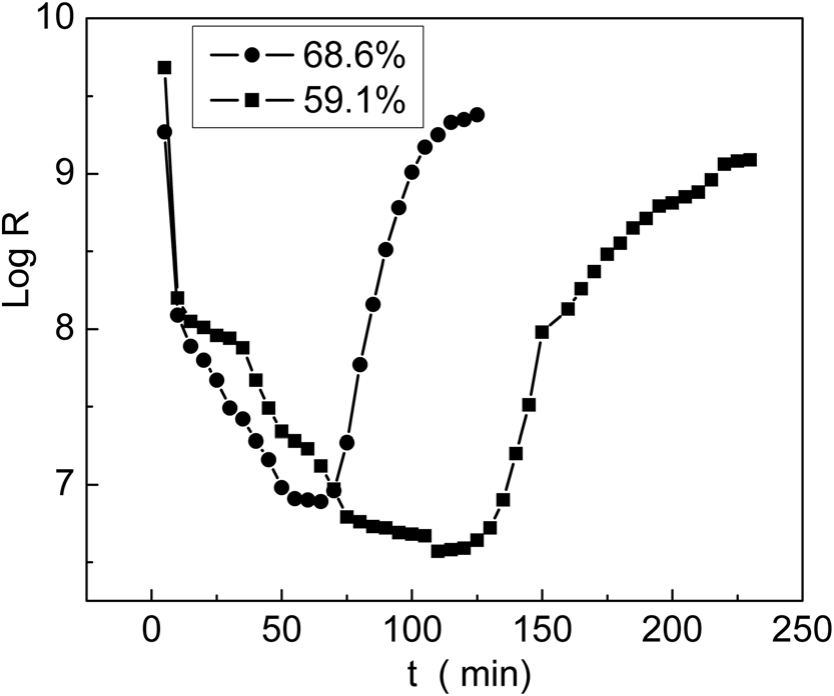
Relationship between log *R* and time at different *G*_t_.

The grafted membrane was first placed in a container with 5% humidity until the resistance reached equilibrium; then, it was transferred to another container with 98% humidity. We found that the resistance decreased sharply in a short time and then leveled off. In the meantime, we recorded the resistance values and obtained the first half of a log *R*–*t* curve, which is called the response curve. Then, the membrane was taken out of the container with 98% humidity and placed back into the container with 5% humidity. The membrane resistance rapidly increased and finally reached equilibrium. The second half of the log *R*–*t* curve was named as the recovery curve. The entire curve including the response and recovery processes is shown in Fig. 12; we can see that the membrane with 68.6% grafting yield responded and recovered more rapidly than the membrane with 59.1% grafting yield. In comparison with the result reported by Sangthumchai,^39^ the total response and recovery time of our prepared membrane with bifunctional groups –COOH and –SO_3_Na were shorter than those of a membrane that contained only –SO_3_Na groups. Due to weak interactions between the –COOH groups and absorbed H_2_O, the adsorbed H_2_O was easily desorbed; thus, the total response and recovery time became shorter.

## 5. Conclusion

Radiation-grafted cation exchange membranes were prepared by a one-step method to investigate the effect of bifunctional groups on the properties of the membranes. New absorption peaks assigned to C=O and S=O indicated that the monomers AA and SSS were grafted onto ETFE membranes. When the grafting reaction was carried out at 80 °C, the highest grafting rate and *G*_t_ were obtained. The addition of HCl was an effective method for the enhancement of *G*_t_ and *G*_s_. When irradiated ETFE films were kept at −19 °C, the decay rate of trapped radicals was very slow; thus, *G*_t_ dropped from 50.0 to 43.8% during a period of 144 h storage time.

After the introduction of –COOH and –SO_3_Na groups, the grafting membranes not only exhibited excellent hydrophilicity but also showed high alkali uptake values. The resistance of the membranes decreased by 4 orders of magnitude at high humidity and therefore, the grafting membranes can be used as humidity sensitive materials for humidity sensors. The membranes with bifunctional groups displayed shorter response and recovery times compared to the ones containing only –SO_3_H groups. The charging voltage of the grafted membrane was lower than that of a commercial separator that only contained carboxylic acid groups. Furthermore, the charging voltage was still relatively stable after 100 cycles; therefore, the grafting membranes have great potentials to be used as separators in secondary batteries.

## Conflicts of interest

There are no conflicts to declare.

## Supplementary Material
